# Gender differences in non-cystic fibrosis bronchiectasis severity and bacterial load: the potential role of hormones

**DOI:** 10.1177/17534666211035311

**Published:** 2021-09-14

**Authors:** Anna Brooke-Hollidge, Joy Conway, Adam Lewis

**Affiliations:** Brunel University London, Uxbridge, UK; Brunel University London, Uxbridge, UK; College of Health, Medicine and Life Sciences, Brunel University London, Mary Seacole Building, Kingston Lane, Uxbridge, UB8 3PH, UK

**Keywords:** bacterial load, bronchiectasis, gender, hormones, menopause, NCFB, physiotherapy, HRT

## Abstract

Non cystic-fibrosis bronchiectasis (NCFB) is a complex chronic respiratory disease, characterised by excessive sputum production and abnormal permanent dilation of bronchi. Mucus accumulation leads to recurrent bacterial infections and increased bacterial load, causing vicious cycles of structural damage and decreased lung function. Respiratory physiotherapy management of NCFB includes airway clearance techniques and use of nebulised, hypertonic saline. Despite advances in treatment, a consistent relationship has been observed between gender and disease occurrence, with a higher prevalence amongst females. Furthermore, NCFB presents most aggressively amongst post-menopausal females, a group likely exposed to higher levels of progesterone (P4) over a longer period of time. The effects of gender-specific hormones on bacterial load and physiotherapy management of people living with NCFB remain unknown. The aim of this narrative review was to discuss the potential influence of gender specific hormones on NCFB disease progression and influence on physiotherapy, medical management and future research. SCOPUS and PUBMED electronic databases were used to conduct searches for relevant studies using specific inclusion and exclusion criteria. Secondary inclusion of relevant literature was obtained from primary paper references. Previous literature suggests that P4 may impair Cilia Beat Frequency (CBF) in airway epithelium. Reduction in CBF may further reduce ability to expectorate amongst individuals with NCFB, increasing bacterial load and likelihood of exacerbations, negatively impacting on disease progression. Furthermore, coadministration of Estrogen has been suggested to offer opposing effects to that of P4 only. These findings question whether hormonal levels may be monitored, controlled and optimised within management and treatment of females with NCFB to improve airway clearance, reduce exacerbations and improve quality of life. Larger scale, long-term trials are required to further explore the effects of gender specific hormones on NCFB and the viability of treatment with hormone replacement therapy.

*The reviews of this paper are available via the supplemental material section*.

## Introduction

This narrative review explores the role of gender in the pathogenesis and management of non-cystic fibrosis bronchiectasis (NCFB). The potential role of hormones in the increased prevalence of NCFB amongst females will be discussed. Specifically, the reduction of cilia beat frequency (CBF) as a result of increased progesterone (P4) levels, the implications for physiotherapy management and future research indications.

## Search strategy

The electronic databases SCOPUS and PUBMED were used to conduct systematic searches for relevant studies. The following search terms were used, with synonyms and closely related words: ‘non-cystic fibrosis bronchiectasis’, ‘hypertonic saline’, ‘microbiology’, ‘gender’, ‘hormone’, ‘menopause’ and ‘bacterial load’. Further studies were identified by examining the reference lists of included articles. Searches were not limited by hypertonic saline (HTS) solution concentration or study type. Studies meeting the following criteria were excluded: non-English language, published >10 years ago, not available *via* the Brunel Library database and those referring only to paediatric populations.

## Bronchiectasis

Bronchiectasis is a complex multifactorial chronic respiratory disease (CRD) characterised by the abnormal, permanent, and irreversible dilation of individual or multiple bronchi. Dominant features include airway inflammation, a chronic cough and excessive sputum production.^1,2^ Mucus accumulation leads to recurrent infections and bronchial damage, feeding a vicious cycle of further infections and decreased lung function. This has significant negative impacts on quality of life (QoL).^1^ Recent literature demonstrates that morbidity and mortality from NCFB is increasing.^3^ NCFB refers to bronchiectasis which is either idiopathic in nature or provoked by non-cystic fibrosis (CF) related respiratory disorders. Exacerbations of NCFB account for a large proportion of the clinical workload and economic impact on health care systems internationally.^4^

## Bacteria

Bacterial presence is an important factor to consider in NCFB disease management. Literature shows that individuals with no pathogens present in sputum samples have the mildest form of disease.^5^ Bacterial load may be defined as a marker for the degree of chronic bacterial airway infection within the lungs, reported as change in colony-forming units per gram (cfu/g) of sputum.^6^ Previous culture studies demonstrate a consistent relationship between bacterial load and airway inflammation using both sputum and bronchial wash as measures.^7–9^ Furthermore, airway inflammation is linked directly to recurring exacerbations, disease progression and higher rates of mortality.^10^

## Pseudomonas

Existing data exploring the bronchiectasis microbiome highlights the frequent domination of lung bacterial communities by *Pseudomonas*, *Haemophilus* and *Streptococcus. Pseudomonas* and *Haemophilus* dominated microbiomes have previously been linked to increased disease severity and exacerbation frequency.^10^ Although data into microbiomes is currently limited for NCFB, colonisation of *Pseudomonas* amongst adults with bronchiectasis is also associated with increased risk of hospital admissions, exacerbations and an approximately threefold increase in risk of mortality.^11^

## Non-tuberculous mycobacteria

Multiple bacterium may be detected within sputum samples, including non-tuberculous mycobacteria (NTM), which are environmental microbes that are potentially pathogenic in nature. A global rise has been demonstrated in the incidence of NTM infections, particularly amongst postmenopausal women.^12^ Multiple risk factors have been identified for NTM colonisation, including NCFB. Furthermore, literature has demonstrated a higher probability of NTM isolation amongst elderly females with a low body mass index.^13^ British Thoracic Society (BTS) guidelines for the medical management of bronchiectasis suggest routine 6-monthly monitoring of sputum culture, unless deterioration is observed.^14^ For NCFB, current research exists to support the use of nebulised 7% HTS alongside physiotherapy in the management of sputum expectoration and QoL.^1^ However, the effects of these treatments on bacteria have not been explored thoroughly.

## Gender

In addition to bacterial load, gender differences in NCFB are clinically apparent and important to consider.^2^ Bronchiectasis is estimated to be prevalent in 566/100,000 women and 485/100,000 men in the United Kingdom (UK).^4^ In addition to increased incidence, literature demonstrates women to have increased disease severity, poorer lung function and a survival disadvantage compared with males across all age groups.^15–17^ It was initially thought that this disadvantage was due to social norms, such as expectoration in public and anatomical differences. Women have smaller lungs and therefore smaller conducting airways – a feature associated with predisposition to infection.^18^ However, it has since been suggested that the pathophysiology of bronchiectasis may also be influenced by other factors, including gender-specific hormones.^2^

## Hormones

### Oestradiol

Previous CF literature indicates earlier conversion to mucoid strains amongst women colonised with *Pseudomonas aeruginosa* than men.^16^ This has been suggested to be due to the presence of the female specific hormone oestradiol (E_2_), which, during periods of high circulating levels, is associated with diminished sol layer and reduced inflammatory response to agonists.^19^ Findings from a recent clinical study demonstrated E_2_ to be significantly associated with selectivity for mucoid isolation, increased exacerbations and mucoid conversion *in vivo*. Furthermore, a significant relationship between exacerbation occurrence and E_2_ levels according to day of menstrual cycle was observed. Most exacerbations occurred during the follicular phase, when E_2_ levels are at their highest.^20^

### Progesterone

Bronchiectasis has been observed to present most aggressively in post-menopausal women.^2^ Further research is required to investigate the driving force behind this, and gender differences consistently observed in NCFB literature.

Mucociliary clearance (MCC) is an important respiratory defence mechanism that aids in the clearance of potentially harmful foreign particles, pathogens and gaseous materials from the airways to maintain healthy lungs.^21^ The epithelial surface of the respiratory tract is lined with ciliated cells, a protective mucous layer and a liquid sol layer in which cilia may move effectively. Cilia are specialised hair-like structures, which, in health, beat in coordinated metachronal waves to propel inhaled particles trapped in the mucous layer out of the airways, towards the mouth where they may be expectorated or swallowed.^22^ Cilia beat frequency (CBF) is a key measurable factor determining the rate of MCC and may be regulated by multiple physiological factors.^21^ Reduced CBF is associated with chronic respiratory disease.^22^

Previous studies of gender-specific hormones on oviduct epithelium have revealed P4 to inhibit CBF.^23,24^ In one study by Paltieli *et al.*, fallopian tube epithelial samples were incubated in media containing a variety of hormones and concentrations.^24^ Samples were then analysed against control samples according to CBF, measured using a photoelectric technique. At 24 h post addition of P4 in concentrations of 0.5 or 1 ng/ml, a significant reduction of CBF was observed (63% of the control levels). Furthermore, in samples where the concentration of P4 was equal to or above 2 ng/ml, 50–70% of cilia were paralysed. Alternatively, when re-incubated with E_2_, effects of P4 were opposed, with a 4% increase in mean CBF.

Given the common ciliated surface of airway and fallopian tube epithelium,^25^ findings from previous studies of ciliated epithelial cells have prompted investigation of further similarities. Jain *et al.* evaluated P4 receptor (PR) expression in human lung and cultured human airway epithelial cells from male and female lung transplant donors.^26^ Primary human tracheal epithelial cells (hTECs) were imaged at 37°C on membranes using an inverted, high-contrast microscope. CBF was captured using a high-speed camera and interpreted using a video analysis system. Findings demonstrated P4 to inhibit CBF in the airway epithelium of both mice and human lung donors.^26^ In this study, exposure to P4 decreased CBF by 42.3%. Interestingly, coadministration of P4 with the active form of oestrogen, 17b-estradiol, or the PR antagonist mifepristone, prevented inhibition of CBF. Despite the findings discussed, no studies currently exist in exploration of the effects of P4 and oestrogen on CBF, sputum expectoration and bacterial load amongst people living with CRD.

Overall, serum P4 ranges are higher and more variable amongst females than males. This is in accordance with point in menstrual cycle, pregnancy and perimenopause. Post menopause, levels of P4 drop to a consistent and relatively low level of 1 ng/dl or less, equal to males.^27^

#### Normal serum Progesterone ranges

**Figure 1.**
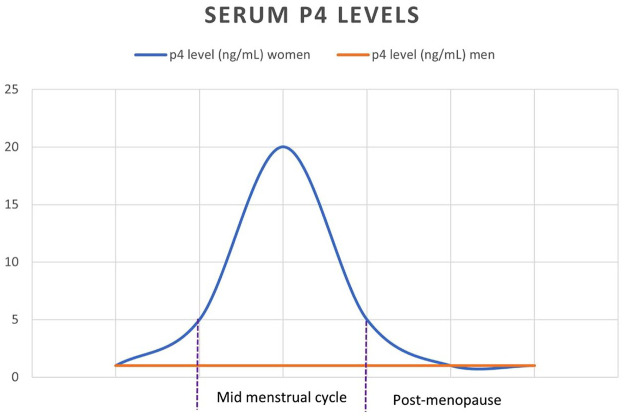
Serum P4 levels in men and women. P4, progesterone.

Normal serum progesterone ranges are^27^:

- men, post-menopausal women, and women at the beginning of their menstrual cycle: 1 ng/ml or under (Figure 1);
- women in the middle of their menstrual cycle: 5–20 ng/ml;
- pregnant women in their first trimester: 11.2–90 ng/ml;
- pregnant women in their second trimester: 25.6–89.4 ng/ml;
- pregnant women in their third trimester: 48.4–42.5 ng/ml.

If P4 does reduce CBF in airways, then, despite fluctuations, in comparison with males, females may spend a higher proportion of their lifetime with reduced CBF, and increased bacterial loads. Therefore, despite permanent reduction in P4, extensive physiological damage may have occurred amongst post-menopausal females, potentially more so than in males. This would not only explain prevalence gender differences observed in research to date, but also the disadvantageous emphasis placed on post-menopausal women.

The same has been found in pregnant females, where P4 levels also increase.^28^ In such circumstances, where there is a rise in P4, literature has found CBF to demonstrate improvements when exposed to additional oestrogen.^26^ Pregnancy zone protein (PZP) is a high-molecular-weight glycoprotein that is elevated in the serum of pregnant women and the synthesis of PZP is oestrogen dependent.^28^ Finch *et al.* performed a validation study of 124 patients with NCFB to characterise PZP in airways and explored its relationship with disease severity.^28^ Sputum protein profiling was achieved using label-free liquid chromatography and mass spectrometry. PZP levels and airway infection status were then measured by use of the validated enzyme-linked immunosorbent assay (ELISA) and 16S ribosomal RNA sequencing, retrospectively. Results demonstrated that sputum, but not serum concentrations of PZP, were associated significantly with the bronchiectasis severity index, the frequency of exacerbations and symptoms. Samples were then compared between patients with and without *P. aeruginosa* present. Airway infection with Proteobacteria such as *P. aeruginosa* was associated with higher concentrations of PZP. Sputum PZP levels were also related directly to airway bacterial load.^28^

### Oestrogen

During pregnancy, large amounts of mucoid fluid are produced in response to cervical stimulation by P4 and oestrogen. This fills the endocervical canal, forming a mucous plug known as the operculum. The operculum acts as a barrier to prevent against infection during pregnancy.^29^ Previous studies using microrheometry to measure the viscoelasticity of human cervical mucus samples also demonstrate increases in mean viscosity during the ovulatory phase of the menstrual cycle.^30^ To our knowledge, the effects of ovulation and pregnancy on sputum viscosity and rate of production have not yet been examined in human airways. However, previously discussed literature demonstrates multiple shared features of ciliated epithelial cells within the human oviduct and airway. Therefore, it may be that females are not only more frequently exposed to reduced CBF, but may also be subject to regular/monthly changes in sputum production with regards to volume and viscosity, potentially impairing airway clearance further.

With regards to oestrogen, previous literature demonstrates elevations in oestrogen amongst females with CF to be associated with inhibited Cl^–^ secretion, causing reduced mucus clearance for approximately 1 week per month.^19^ This further increases susceptibility to acute exacerbations and contributes to gender differences observed amongst CF patients.^19^ Another study into the effects of hormones on a female CF population demonstrated a significant increase in sputum pro-inflammatory cytokines during ovulation, where oestrogen peaks. A decrease in lung function was also observed during this time frame.^31^ Further research is required to examine the combined effects of oestrogen and P4 on inflammation and susceptibility to exacerbation, taking into account their naturally fluctuating levels and those optimum for CBF.

## Management of NCFB

### Airway clearance techniques

Management of NCFB places a heavy emphasis on airway clearance techniques (ACTs), which aim to mobilise secretions, aiding effective expectoration and airway clearance.^14^ This increases ventilation efficiency, reducing dyspnoea, thoracic pain, and further daily sputum production.^32^ ACTs are therefore important to consider in the wider context of therapies offered to patients in multidisciplinary care. Given the potentially negative effects of P4 on CBF, and monthly increase in sputum volume and viscosity discussed, it may be pragmatic to increase the frequency of such therapies amongst women according to point in menstrual cycle, hormonal status and pregnancy.

### Hypertonic saline

In addition to ACTs in the management of NCFB, nebulised HTS is becoming increasingly used within respiratory physiotherapy.^33^ HTS is a sterile, saline solution of different concentrations higher than that of isotonicity, which may aid in sputum clearance by increasing ciliary function and osmotic drive, whilst reducing sputum viscosity by altering its visco-elastic properties.^33,34^ Literature examining the effects of HTS as an adjunct to physiotherapy amongst individuals with NCFB demonstrate improvements in forced expiratory volume in 1 second (FEV1), sputum weight, sputum viscosity, ease of expectoration and self-reported QoL.^1,34,35^ Benefits have also been reported in clinical practice.^36^ The use of HTS in NCFB is now supported in BTS guidelines.^14,32^

HTS may be delivered in a variety of salt concentrations, the most effective of which remains unestablished.^37^ Amongst CF populations, literature measuring patient response to differing concentrations of inhaled HTS demonstrates a significant increase in mucociliary clearance post intervention for 0.9%, 3.0%, 7.0%, and 12% HTS. Results suggest effects of HTS to be dose dependent, with increased doses corresponding to increased clearance.^38^ If such findings translate to NCFB, it may be that HTS use and dosage should be altered according to patient hormonal status to maintain optimum CBF. For example, increasing concentration and frequency of HTS administration at times when P4 levels are known to be increased, such as pregnancy and ovulation. However, the increasing risks of adverse events such as bronchoconstriction and exacerbation of symptoms should be noted with increasing doses.

### Anti-inflammatories

As previously stated, airway inflammation is linked directly to recurring exacerbations, disease progression and mortality.^10^ Therefore, anti-inflammatories are frequently used in the management of NCFB. These commonly include macrolides and antibiotics.^39^

Macrolides have been shown to have both antimicrobial and anti-inflammatory effects.^39^ Trials demonstrate a significant reduction in exacerbation frequency amongst those receiving macrolide therapy compared with placebo groups.^39–42^ However, macrolides are also associated with a potential increase in resistance to oropharyngeal and other bacteria, typically the previously discussed NTM.^39,40^ This would be problematic in the treatment of post-menopausal women with NCFB.

Prospective studies have also demonstrated the administration of ovarian hormones to postmenopausal women to modulate surrogate markers of vascular inflammation. Significant decreases were observed in E-selectin, sVCAM1, sICAM-1, and TNF-α. In contrast, oral hormone therapy increases CRP.^43^ Although the impact of this effect has been disputed, it may be that oestrogen has the potential to act as an anti-inflammatory.

### Inhaled antibiotics

The use of antibiotic therapy has been indicated to reduce airway inflammation, allow airway healing and delay disease progression.^7^ Although multiple data regarding CF-bronchiectasis exist to support this, data surrounding NCFB are limited.^44^ A 12-month controlled trial of nebulised gentamicin amongst an NCFB population demonstrated high airway bacterial loads to be associated with airway and systemic inflammation and a greater risk of exacerbations. Both short- and long-term antibiotic therapy were found to reduce markers of airways and systemic inflammation.^7^

### Implications for physiotherapy practice and research

Limited literature exists to address the impact of gender on response to treatment in NCFB.^26^ Since HTS is thought to increase ciliary function,^34^ exploration into the effects of HTS on bacterial load in males *versus* females and pre-menopausal *versus* post-menopausal individuals may be indicated. Furthermore, if gender difference in NCFB is related to hormones, this may lead to the generation of new, specific and individually tailored physiotherapy advice. Such advice may extend to pregnancy, where patients may find it more difficult to clear sputum even with HTS use.

Literature suggests that inhibition of CBF may be prevented if oestrogen and P4 are present in combination.^26^ However, CBF improvement would be highly time dependent. Dual presence occurs only in younger women, decreases over time, fluctuates during monthly ovulation and is dependent on birth control methods. During times where there is likely an increase in P4, such as pregnancy and during menstrual cycles, it may be that frequency of physiotherapy should be increased. Furthermore, if HTS is equally effective for males and females, it may be that females would also benefit from a higher dose or increased frequency of administration as an adjunct to physiotherapy.

In the interest of multidisciplinary team working, recommendations might also extend to advice on suitable methods of birth control, and referrals to medical teams regarding potential administration of hormone replacement therapy (HRT). HRT is commonly used to avoid undesirable effects of menopause. However, it must be noted that HRT is not without contraindications, and therefore may not be offered to all individuals.^45^ Furthermore, due to the adverse effects of oestrogen on airway secretion hydration in CF populations, anti-oestrogen therapies have been proposed previously.^19^ This should also be considered.

It is not yet clear for which age category or gender coadministration of P4 and oestrogen may be most beneficial. Post-menopausal females experience reduction not only in P4, but also in oestrogen levels. Additionally, factors such as genetics, over-the-counter supplements, and a wide variety of methods of contraception must be considered. Registry data within previous CF literature has demonstrated that, when prescribed oral contraceptives, females have lower rates of exacerbation, requiring less use of antibiotics.^20^ Furthermore, in a CF population, fluctuations in sputum inflammatory biomarkers improved when hormonal contraception use was used.^31^

## Conclusion

NCFB is a CRD associated with increased mucus accumulation and expectoration difficulty – a mechanism which is linked directly with CBF. NCFB is more common amongst females, presenting most aggressively post-menopause. Until post-menopause, females display fluctuating increased P4 levels compared with males. Literature examining airway epithelium of human lung donors has previously demonstrated P4 to impair CBF. Although not yet investigated, if this is also the case amongst the living, then this reduction in CBF is likely to reduce ability to expectorate, thus increasing bacterial load and likelihood of exacerbations, negatively impacting disease progression amongst individuals with NCFB. Previous literature also demonstrates coadministration of oestrogen to offer opposing effects to that of P4 alone on CBF. However, its potential role in the medical management of females with NCFB in order to increase CBF and sputum expectoration remains unexplored. Although further research is required, previous literature suggests that HRT may be a beneficial therapeutic intervention for post-menopausal women to improve airway clearance, reduce exacerbations and improve QoL. Gender specific hormones have the potential to be of great importance in directing future management of NCFB. Adjusted frequency and type of physiotherapy intervention according to patient age, gender and hormonal status may be required. Furthermore, physiotherapy and medical management may also extend to advice regarding birth control methodology, HRT and planned, medically overseen, pregnancy. Further trial data are now urgently needed.

## Supplemental Material

sj-pdf-1-tar-10.1177_17534666211035311 – Supplemental material for Gender differences in non-cystic fibrosis bronchiectasis severity and bacterial load: the potential role of hormones Click here for additional data file. Supplemental material, sj-pdf-1-tar-10.1177_17534666211035311 for Gender differences in non-cystic fibrosis bronchiectasis severity and bacterial load: the potential role of hormones by Anna Brooke-Hollidge, Joy Conway and Adam Lewis in Therapeutic Advances in Respiratory Disease

sj-pdf-2-tar-10.1177_17534666211035311 – Supplemental material for Gender differences in non-cystic fibrosis bronchiectasis severity and bacterial load: the potential role of hormones Click here for additional data file. Supplemental material, sj-pdf-2-tar-10.1177_17534666211035311 for Gender differences in non-cystic fibrosis bronchiectasis severity and bacterial load: the potential role of hormones by Anna Brooke-Hollidge, Joy Conway and Adam Lewis in Therapeutic Advances in Respiratory Disease

sj-pdf-3-tar-10.1177_17534666211035311 – Supplemental material for Gender differences in non-cystic fibrosis bronchiectasis severity and bacterial load: the potential role of hormones Click here for additional data file. Supplemental material, sj-pdf-3-tar-10.1177_17534666211035311 for Gender differences in non-cystic fibrosis bronchiectasis severity and bacterial load: the potential role of hormones by Anna Brooke-Hollidge, Joy Conway and Adam Lewis in Therapeutic Advances in Respiratory Disease
