# Supplementary material for: Gender differences in non-cystic fibrosis bronchiectasis severity and bacterial load: the potential role of hormones
Source: Ther Adv Respir Dis. 2021 Sep 14;15:17534666211035311. doi: 10.1177/17534666211035311 (PMC8445533; doi:10.1177/17534666211035311)
Supplement: sj-pdf-2-tar-10.1177_17534666211035311 – Supplemental material for Gender differences in non-cystic fibrosis bronchiectasis severity and bacterial load: the potential role of hormones [file sj-pdf-2-tar-10.1177_17534666211035311.pdf]

Reviewer 1 v.1

Comments to the Author

This is a thought provoking review article addressing the gender differences observed among people with non-CF bronchiectasis.

The authors give us an overview of the management of bronchiectasis with physiotherapy and hypertonic saline and then move into the possible impact of hormones on cilia beat frequency and how this may effect the sex differences among people with non-CF bronchiectasis.

Major concerns:

- While this is a nice summary, the organization of the article was very challenging to follow.

The article began with overview of bronchiectasis and the treatments, which may not be relevant to this manuscript as none of these were put in the context of gender specific effects.

- The authors have a paragraph on page 7 directed at bacteria, but do not discuss inhaled antibiotics and their role in bronchiectasis or data that exists in CF about the increase impact of Pseudomonas in people with CF. In addition, they do not discuss the higher prevalence of nontuberculous mycobacteria in women with non-CF bronchiectasis.

- Also, within the management strategies, there is no discussion of the role of inflammation and use of anti-inflammatories in non-CF bronchiectasis. This is a critical part of the disease process in bronchiectasis and has data to support the role of hormones impacting this aspect of disease.

There are many articles within the CF space about the gender disparity in regards to airway mucus, inflammation and bacteria. Examples: Chotirmall et al. N Engl J Med 2012; 366: 1978-1986.

Coakley et al. JCI. 2008; 118 : 4025-4035.

Holtrop et al. Ann Am Thorac Soc. 2021. While the authors may not have intended to discuss CF, they site multiple CF references and talk about gender based survival differences in bronchiectasis on page 6, but all of these references were based on data in CF.

- The authors describe their methods in the abstract and on page 5 under search strategy, but do not describe how many articles this search revealed and how they selected the ones to discuss.

- There are articles in the CF discussing the potentially beneficial impact of OCP on exacerbations and inflammation. It may be beneficial to reference these (Chotirmall et al. NEJM 2012; Holtrop et al. Ann Am Thorac 2021)

Minor concerns:

- Abstract – not sure that chronic respiratory disease should be capitalized; not sure physiotherapy in respiratory physiotherapy should be capitalized

- Abstract conclusion – first sentence may need some rewording: "…previous findings suggest P4 to impair CBF…"

- Page 6 – BTS is used for the first time without defining British Thoracic Society

- Page 7 – BTS is used for the second time and defined here

- Page 7 – under bacteria section: "…medical management of Bronchiectasis suggest six-monthly monitoring of sputum culture" – needs to be reworded and bronchiectasis does not need to be capitalized.

- Page 12 – unclear on MDT abbreviation
