# Supplementary material for: Gender differences in non-cystic fibrosis bronchiectasis severity and bacterial load: the potential role of hormones
Source: Ther Adv Respir Dis. 2021 Sep 14;15:17534666211035311. doi: 10.1177/17534666211035311 (PMC8445533; doi:10.1177/17534666211035311)
Supplement: sj-pdf-3-tar-10.1177_17534666211035311 – Supplemental material for Gender differences in non-cystic fibrosis bronchiectasis severity and bacterial load: the potential role of hormones [file sj-pdf-3-tar-10.1177_17534666211035311.pdf]

Reviewer 2 v.1

Comments to the Author

In this review authors aimed to discuss the potential influence of gender-specific hormones on Non Cystic-Fibrosis Bronchiectasis (NCFB) disease progression and influence on physiotherapy, medical management and future research. The authors included various databases and included extensive literature to describe the role of gender-specific hormones on bacterial load and physiotherapy management of people suffering from NCFB. Further, the authors conclude that hormonal levels in males and females play a crucial role in regulating NCFB and monitoring or controlling the hormonal levels could be used to manage and treat NCFB to improve airway clearance, reduce exacerbations and improve quality of life, especially in females. Overall, conceptually manuscript provides novel information for the improvement of avenues to the scientific community in the context of gender and occurrence of NCFB. However, the manuscript has the following few points that need to address before consideration.

1. The authors provided a good correlation for NCFB occurrence in females and progesterone level. However, the mechanistic basis of progesterone and its role in regulating NCFB is missing. It would be good for readers if authors can provide a detailed mechanism probably with a graphical representation.

2. The authors highlighted progesterone in the review however provided very brief details about estrogen and its role in NCFB. Given the primary sex hormone in females, it would be good if authors can explore more about estrogen and its role in NCFB.

3. AS per the recent research and also mentioned in the review, NCFB is more common amongst females than males, in this case, is testosterone which is the primary sex hormone in males has any protective effect against NCFB. Please explain.

4. The authors did not mention anything about the pre-puberty age stage and NCFB in both genders.
