# Supplementary material for: Gender differences in non-cystic fibrosis bronchiectasis severity and bacterial load: the potential role of hormones
Source: Ther Adv Respir Dis. 2021 Sep 14;15:17534666211035311. doi: 10.1177/17534666211035311 (PMC8445533; doi:10.1177/17534666211035311)
Supplement: sj-pdf-1-tar-10.1177_17534666211035311 – Supplemental material for Gender differences in non-cystic fibrosis bronchiectasis severity and bacterial load: the potential role of hormones [file sj-pdf-1-tar-10.1177_17534666211035311.pdf]

Author Response

Response to Reviewer Comments

Manuscript ID TAR-21-032 entitled "Gender Differences in Non-Cystic Fibrosis Bronchiectasis Severity and Bacterial Load: The Potential Role of Hormones"

Thank you to the reviewers for giving such good feedback in order for us to improve our manuscript further. We are very happy that you feel our work is of value to the publication. Please see below your original comments and our responses below with indication where in the re-submitted manuscript the changes can be seen. We hope that you find the revised manuscript worthy of publication.

REVIEWER 1:

Major concerns:

1) While this is a nice summary, the organization of the article was very challenging to follow.

The article began with overview of bronchiectasis and the treatments, which may not be relevant to this manuscript as none of these were put in the context of gender specific effects.

Thank you, this feedback is much appreciated, we want the reader to be able to follow the article as easily as possible.

We have now rearranged the order to place current treatments section at end, noting that these other treatments focus on airway clearance which are therefore important to consider in the wider context of therapies offered to patients in MDT delivered care, and therefore we may need to investigate increasing the frequency of such therapies according to point in menstrual cycle/hormonal status/pregnancy etc. This is the same for HTS in relation to increasing CBF.

2) The authors have a paragraph on page 7 directed at bacteria, but do not discuss inhaled antibiotics and their role in bronchiectasis or data that exists in CF about the increase impact of Pseudomonas in people with CF. In addition, they do not discuss the higher prevalence of nontuberculous mycobacteria in women with non-CF bronchiectasis.

Thank you for this. We have now included a section on antibiotics and their role in bronchiectasis on page 13. Which states:

“Inhaled antibiotics:

The use of antibiotic therapy has been indicated to reduce airway inflammation, allow airway healing and delay disease progression. 7 Although multiple data regarding CF-Bronchiectasis exists

to support this, data surrounding NCFB is limited 45. A 12-month controlled trial of nebulised gentamicin amongst an NCFB population demonstrated high airway bacterial loads to be associated with airway and systemic inflammation and a greater risk of exacerbations. Both short- and long-term antibiotic therapy were found to reduce markers of airways and systemic inflammation. 7 "

We have also now included a section/s on data that exists on the impact of pseudomonas in people with CF on pages 4-5. Which states:

"Pseudomonas:

Existing data exploring the Bronchiectasis microbiome highlights the frequent domination of lung bacterial communities by Pseudomonas, Haemophilus and Streptococcus. Pseudomonas and Haemophilus dominated microbiomes have previously been linked to increased disease severity and exacerbation frequency 10. Although data into microbiomes is currently limited for NCFB, colonisation of Pseudomonas amongst adults with Bronchiectasis is also associated with increased risk of hospital admissions, exacerbations, and an approximately threefold increase in risk of mortality. 11"

We have also now included a section/s on data that exists on the impact of NTM on page 5. Which states:

"Non-tuberculous mycobacteria:

Multiple bacterium may be detected within sputum samples, including Non-tuberculous mycobacteria (NTM). Non-tuberculous mycobacteria are environmental microbes which are potentially pathogenic in nature. A global rise has been demonstrated in the incidence of NTM infections, particularly amongst postmenopausal women 12. Multiple risk factors have been identified for NTM colonisation, including NCFB. Furthermore, literature has demonstrated a higher probability of NTM isolation amongst elderly females with a low body mass index 13."

3) Also, within the management strategies, there is no discussion of the role of inflammation and use of anti-inflammatories in non-CF bronchiectasis. This is a critical part of the disease process in bronchiectasis and has data to support the role of hormones impacting this aspect of disease.

There are many articles within the CF space about the gender disparity in regards to airway mucus, inflammation and bacteria. Examples: Chotirmall et al. N Engl J Med 2012; 366: 1978-1986.

Coakley et al. JCI. 2008; 118 : 4025-4035.

Holtrop et al. Ann Am Thorac Soc. 2021. While the authors may not have intended to discuss CF, they site multiple CF references and talk about gender based survival differences in bronchiectasis on page 6, but all of these references were based on data in CF.

This is a very important point which has now been included, along with the references suggested – many thanks.

We have now included a section on anti-inflammatories and their role in bronchiectasis on page 12-13. Which states:

“Anti-inflammatories

As previously stated, airway inflammation is directly linked to recurring exacerbations, disease progression and mortality.10 Therefore, anti-inflammatories are frequently used in the management of NCFB. These commonly include macrolides and antibiotics 40.

Macrolides have been shown to have both antimicrobial and anti-inflammatory effects 40. Trials demonstrate a significant reduction in exacerbation frequency amongst those receiving macrolide therapy compared to placebo groups 40 41 42 43. However, macrolides are also associated with a potential increase in resistance to oropharyngeal and other bacteria, typically the previously discussed NTM 40 41. This would be problematic in the treatment of post-menopausal women with NCFB.

Prospective studies have also demonstrated the administration of ovarian hormones to postmenopausal women to modulate surrogate markers of vascular inflammation. Significant decreases were observed in E-selectin, sVCAM1, sICAM-1, and TNF-α. In contrast, oral hormone therapy increases CRP 44. Although the impact of this effect has been disputed, it may be that Estrogen has the potential to act as an anti-inflammatory.”

We have also now included a section on Estradiol and its impact on mucoid conversion/exacerbation on page 6. Which states:

“Estradiol

Previous CF literature indicates earlier conversion to mucoid strains amongst women colonised with Pseudomonas Aeruginosa than men 19. This has been suggested to be due to the presence of the female specific hormone Estradiol (E2), which, during periods of high circulating levels, is asso-ciated with diminished sol layer and reduced inflammatory response to agonists 20. Findings from a recent clinical study demonstrated E2 to be significantly associated with selectivity for mucoid isola-tion, increased exacerbations, and mucoid conversion in vivo. Furthermore, a significant relation-ship between exacerbation occurrence and E2 levels according to day of menstrual cycle was ob-served. Most exacerbations occurred during the follicular phase, where E2 levels are at their high-est 21.“

We have also now included a section on Estrogen. This includes its impact on exacerbation frequency/potential role as an anti-inflammatory on page 10-11. Which states:

“With regards to Estogen, previous literature demonstrates elevations in Estrogen amongst females with CF to be associated with inhibited Cl- secretion, causing reduced mucus clearance for approximately 1 week per month 20. This further increases susceptibility to acute exacerbations and contributes to gender differences observed amongst CF patients 20. Another study into the effects of hormones on a female CF population demonstrated a significant increase in sputum pro-inflammatory cytokines during ovulation, where Estrogen peaks. A decrease in lung function was also observed during this time frame 32. Further research is required to examine the combined effects of Estogen and P4 on inflammation and susceptibility to exacerbation, taking into account their naturally fluctuating levels and those optimum for CBF.”

4) The authors describe their methods in the abstract and on page 5 under search strategy, but do not describe how many articles this search revealed and how they selected the ones to discuss.

Thank you for this. We have not included a prisma flow chart because this is a narrative review due to the broad scope of the review. This work was performed as part on an MSc dissertation project, so required the first author to review all articles initially independently. The other authors then inputted further articles of importance/relevance outside of the first authors search when writing up for publication.

5) There are articles in the CF discussing the potentially beneficial impact of OCP on exacerbations and inflammation. It may be beneficial to reference these (Chotirmall et al. NEJM 2012; Holtrop et al. Ann Am Thorac 2021)

Thank you for this – the suggested references have been included within the references section and on page 15:

"Registry data within previous CF literature has demonstrated that, when prescribed oral contraceptives, females have lower rates of exacerbation, requiring less use of antibiotics 21. Furthermore, in a CF population, fluctuations in sputum inflammatory biomarkers improved when hormonal contraception use was used 32."

"32.

Holtrop M, Heltshe S, Shabanova, V, et al. A prospective study of the effects of sex hormones on lung function and inflammation in women with cystic fibrosis. Annals of the American Thoracic Society, 2021."

"21.

Chotirmall SH, Smith SG, Gunaratnam C, et al. Effect of estrogen on pseudomonas mucoidy and exacerbations in cystic fibrosis. New England Journal of Medicine 2012; 366(21): 1978-1986."

Minor concerns:

6) Abstract – not sure that chronic respiratory disease should be capitalized; not sure physiotherapy in respiratory physiotherapy should be capitalized

Many thanks – the above grammatical errors have been amended on page 1:

"respiratory physiotherapy"

7) Abstract conclusion – first sentence may need some rewording: "…previous findings suggest P4 to impair CBF…"

Thank you for this. The sentence in question has been reworded as follows for easier reading on page 1 (Abstract conclusion):

“Previous literature suggests that P4 may impair Cilia Beat Frequency (CBF) in airway epithelium.”

8) Page 6 – BTS is used for the first time without defining British Thoracic Society

Thank you – this has been amended on page 5:

“British Thoracic Society (BTS)”

9) Page 7 – BTS is used for the second time and defined here

Many thanks – this error has been corrected on page 11:

“The use of HTS in NCFB is now supported in BTS guidelines.”

10) Page 7 – under bacteria section: “…medical management of Bronchiectasis suggest six-monthly monitoring of sputum culture” – needs to be reworded and bronchiectasis does not need to be capitalized.

Many thanks – this has been amended on page 5:

“guidelines for the medical management of bronchiectasis suggest routine six-monthly monitoring of sputum culture, unless deterioration is observed.”

11) Page 12 – unclear on MDT abbreviation

Thank you for highlighting this – multidisciplinary team is no longer abbreviated to MDT, but specified as follows:

Page 11: “ACTs are therefore important to consider in the wider context of therapies offered to patients in multidisciplinary care”

Page 14: “In the interest of multidisciplinary team working”

REVIEWER 2:

1) The authors provided a good correlation for NCFB occurrence in females and progesterone level. However, the mechanistic basis of progesterone and its role in regulating NCFB is missing. It would be good for readers if authors can provide a detailed mechanism probably with a graphical representation.

From what current literature suggests, P4 does not directly regulate NCFB, but has the potential to reduce CBF at certain times for women, thus reducing airway clearance. Since airway clearance is the prime method of management for NCFB, P4 levels may indirectly impact on the regulation and management of NCFB. Further research is required amongst living beings/lung transplant donors to clarify this.

If current work regarding mice, epithelial cells and human lung transplant donors translates over to living humans with NCFB, then:

P4 is thought to inhibit CBF. This is reversed with the co-administration of Estrogen. In times when P4 levels are high and Estrogen levels decrease (a monthly occurrence for women and during pregnancy), individuals with NCFB may be at an increased likelihood of exacerbation, further increasing inflammation, bacterial load. This causes further abnormal dilation of bronchi and structural damage, negatively impacting on disease progression.

Amongst females, if this occurs monthly prior to menopause, there is potential for disadvantage due to prolonged exposure to p4. Therefore, despite a decrease in p4 post-menopause, damage has already occurred, potentially more so than in males.

It may be that through medical and multidisciplinary management, we are able to alter the management of NCFB according to gender and hormonal status. For example, increasing frequency/type of therapy, increasing concentration of HTS solution, use of HRT etc. as discussed in the review.

We have now also provided a graphical representation of changing Serum P4 levels in Figure 1.

2) The authors highlighted progesterone in the review however provided very brief details about estrogen and its role in NCFB. Given the primary sex hormone in females, it would be good if authors can explore more about estrogen and its role in NCFB.

This is a very important point the reviewer has raised – thank you for this. To our knowledge, the role of Estrogen in NCFB has not yet been looked into and therefore was not discussed in depth. We focused on P4 because there was more data surrounding it – what little data surrounding Estrogen that was found has included (that it counteracted reduced CBF when added alongside P4).

The use of anti-inflammatories and Estrogen have now been added to the discussion. However, as we have highlighted, there is very little evidence existing in current literature regarding the role of Estorgen in NCFB. This, in itself, needs to be a focus in future research, given its role as a primary gender specific hormone amongst females.

3) As per the recent research and also mentioned in the review, NCFB is more common amongst females than males, in this case, is testosterone which is the primary sex hormone in males has any protective effect against NCFB. Please explain.

Thank you for bringing this up. This is a very interesting point - one which we did not come across in our search. Although literature consistently points out gender difference, testosterone was not mentioned. Reason for gender difference thought to be anatomy/strength of cough/social norms (specifically not wishing to expectorate in public). The main gender specific hormones currently looked into with actual data remain to be Estrogen, Progesterone and PZP. This should be addressed in future research.

4) The authors did not mention anything about the pre-puberty age stage and NCFB in both genders.

Thank you for this. We have not included this because the search included “menopause”/ “hormones”/“pregnancy” and was therefore primarily focused on the adult population. Additionally, this review uses CF as a comparator to NCFB, which will likely include the majority of bronchiectasis patients under the age of 18.
